# Interaction of Sweet Bran inclusion and corn processing method in beef finishing diets

**DOI:** 10.1093/tas/txae023

**Published:** 2024-02-24

**Authors:** Rebecca L McDermott, Braden C Troyer, Maggie E Youngers, Rick A Stock, Galen E Erickson, Jim C MacDonald

**Affiliations:** Department of Animal Science, University of Nebraska, Lincoln, NE 68583, USA; Department of Animal Science, University of Nebraska, Lincoln, NE 68583, USA; Cargill Branded Feeds, Blair, NE 68008, USA; Department of Animal Science, University of Nebraska, Lincoln, NE 68583, USA; Department of Animal Science, University of Nebraska, Lincoln, NE 68583, USA; Department of Animal Science, University of Nebraska, Lincoln, NE 68583, USA

**Keywords:** beef cattle, corn processing, steam-flaking, Sweet Bran, wet corn gluten feed

## Abstract

The study objective was to determine the effects of corn processing method and Sweet Bran (Cargill, Blair, NE) inclusion in beef finishing diets on performance and carcass characteristics. Four hundred and eighty crossbred yearling steers (363 ± 15 kg) were assigned to a 2 × 3 factorial arrangement of treatments, consisting of two corn processing methods, steam-flaked corn (SFC) or a high-moisture corn: dry-rolled corn blend (HMC: DRC), and three inclusions of Sweet Bran (0%, 20%, or 40% of diet dry matter). Data were analyzed using the MIXED procedure of SAS as a generalized block design with pen as the experimental unit and block as a fixed effect. Dry matter intake increased linearly as Sweet Bran increased in the diet, regardless of corn processing method (*P* < 0.01). A corn processing × Sweet Bran interaction (*P* < 0.01) was observed for feed efficiency (G:F), average daily gain (ADG), and hot carcass weight (HCW).

The G:F of steers fed SFC did not change with increasing Sweet Bran concentrations (*P *= 0.19) and the G:F of SFC-fed steers was 12.4% greater than those fed HMC:DRC without Sweet Bran, but was only 5.3% greater when Sweet Bran was included at 40% (*P *= 0.04). The ADG of steers increased linearly with increasing concentration of Sweet Bran in both SFC and HMC:DRC-based diets. However, the interaction occurred (*P* < 0.01) because ADG increased at a greater rate in HMC:DRC-based diets (1.93 to 2.21 kg/d for 0% and 40% Sweet Bran, respectively) compared to SFC-based diets (2.18 to 2.27 kg/d for 0% and 40% Sweet Bran, respectively;). Accordingly, while the ADG of steers fed SFC was 13% greater than steers fed HMC:DRC without Sweet Bran (*P *< 0.01), there was no difference in ADG due to corn processing method at 40% Sweet Bran (*P* = 0.30). In SFC-based diets, HCW tended to increase from 446 to 455 kg as Sweet Bran increased (*P* = 0.06). In HMC: DRC-based diets, HCW linearly increased from 421 to 449 kg (*P* < 0.01), resulting in similar HCW at 40% Sweet Bran (*P* = 0.28). These data suggest HMC:DRC-based diets are more competitive with SFC-based diets due to similar gains and more similar feed efficiencies when Sweet Bran is fed.

## Teaser

Feeding Sweet Bran in high-moisture corn: dry-rolled corn-based diets, make feedlots without steam-flaking capabilities more competitive with steam-flaked corn-based diets due to similar gains and more similar feed efficiencies.

## Introduction

Feeding Sweet Bran replaces a portion of starch from grain in the diet with energy-dense, highly digestible fiber, steep, and solvent-extracted germ meal in finishing diets, which increases dry matter intake (DMI) and results in greater average daily gain (ADG; Stock et al., 2000). Depending on the corn processing method employed, feeding Sweet Bran may maintain or improve feed efficiency (Scott et al., 2003; Macken et al., 2004). Sweet Bran is shipped from plants in the Midwest to the Southern Plains where steam-flaking is a common corn processing method. Research has evaluated increasing concentrations of Sweet Bran in steam-flaked corn (SFC) based finishing diets, but cattle performance responses have differed among studies. Macken et al. (2004) observed similar final weights, ADG, and feed efficiency across treatments (0%, 10%, 20%, 25%, 30%, and 35% Sweet Bran) suggesting up to 35% Sweet Bran can be fed in SFC-based finishing diets. However, Block et al. (2005) observed a quadratic response in final weights, ADG, and feed efficiency when feeding 4 concentrations of Sweet Bran (0%, 20%, 30%, and 40%) suggesting the optimal inclusion of Sweet Bran was between 20% and 30%. Additionally, Domby et al. (2014) observed a quadratic response for final weight and feed efficiency when 0%, 15%, and 30% Sweet Bran were fed, suggesting 15% to 20% is the optimal inclusion.

While steam-flaking is becoming more popular in the Midwest, high-moisture corn (HMC), dry-rolled corn (DRC), and combinations of HMC and DRC are still commonly used (Vasconcelos and Galyean, 2007; Samuelson et al., 2016). Previous research by Scott et al. (2003) and Buckner et al. (2007) has evaluated HMC and DRC-based finishing diets comparing 0% Sweet Bran to only one other Sweet Bran inclusion and observed a linear improvement in DMI, ADG, and feed efficiency when Sweet Bran was included in the diet.

While previous research has evaluated Sweet Bran in SFC and HMC: DRC-based diets separately, a direct comparison of Sweet Bran in SFC and HMC: DRC-based diets has not been evaluated. Based on previous research, we hypothesized a quadratic response for gain and feed efficiency that is maximized at 20% Sweet Bran in SFC-based diets and a linear response for gain and feed efficiency that is maximized at 40% Sweet Bran in HMC: DRC-based diets. The objective of this experiment was to evaluate the interaction between increasing the concentration of Sweet Bran and corn processing method.

## Materials and Methods

All animal-use procedures were reviewed and approved by the Institutional Animal Care and Use Committee (protocol #1785) at the University of Nebraska-Lincoln.

This study was initiated on June 3, 2020 and steers were fed for an average of 161 d. Four hundred and eighty crossbred yearling steers (initial BW = 363 ± 15 kg) were delivered to the University of Nebraska Lincoln’s Eastern Nebraska Research, Extension, and Education Center (ENREEC) feedlot near Mead, NE in October/November of 2019. Steers were processed after arrival, including (1) individual identification and horn tipping as needed; (2) vaccination with a modified live vaccine for prevention of infectious bovine rhinotracheitis, bovine viral diarrhea, parainfluenza-3 (PI_3_), bovine respiratory syncytial virus, *Mannheimia haemolytica*, and *Pasteurella multocida* (Vista Once, Merck Animal Health, De Soto, KS), a killed vaccine for clostridial toxoids and Histophilus somnus (Ultrabac 7/Somubac, Zoetis Inc, Florham Park, NJ); (3) treatment for internal and external parasites (Dectomax, Zoetis Inc.) Approximately 21 d following initial vaccination, steers were revaccinated for *Heamophilus somnus* (Somubac, Zoetis, Inc.) and infectious bovine rhinotracheitis, bovine viral diarrhea, parainfluenza-3 (PI_3_), bovine respiratory syncytial virus, and leptospirosis (Vista 5, Merck Animal Health). Steers were wintered on corn stalks from November 2019 to March 2020 and then held on grass until trial initiation in June 2020.

Steers were limit-fed a common diet consisting of 50% Sweet Bran and 50% alfalfa hay (DM basis) for 5 d at 2% of BW before weighing to minimize gut fill effects and achieve an accurate initial body weight (Watson et al., 2013). Steers were individually weighed using a hydraulic squeeze chute (Silencer, Moly Manufacturing Inc.; Lorraine, KS: scale readability ± 0.90 kg) for 2 consecutive days for initial BW determination (Stock et al., 1983). Steers were blocked by BW into light, medium, and heavy BW blocks (*n* = 4, 3, and 1 replicate(s), respectively) based on first day BW, stratified by BW within block, and randomly assigned to pen within block. Pens within block were assigned randomly to 1 of the 6 treatments, with a total of 10 steers per pen and 8 replications per treatment.

All steers were implanted with 80 mg of trenbolone acetate and 16 mg of estradiol (Revalor-IS; Merck Animal Health; Madison, NJ) 2 d before experiment initiation and given an oral dose of dewormer for control of internal parasites (Safeguard, Merck Animal Health). On days 75 and 76 of the trial, steers were reimplanted with 200 mg of trenbolone acetate and 20 mg estradiol (Revalor-200; Merck Animal Health).

Dietary treatments were arranged in a 2 × 3 factorial, and included (1) 80% HMC: DRC blend with 0% Sweet Bran (HMC:DRC 0), (2) 60% HMC:DRC blend with 20% Sweet Bran (HMC:DRC 20), (3) 40% HMC:DRC blend with 40% Sweet Bran (HMC:DRC 40), (4) 80% SFC with 0% Sweet Bran (SFC 0), (5) 60% SFC with 20% Sweet Bran (SFC 20), and (6) 40% SFC with 40% Sweet Bran (SFC 40). Steers were adapted to the finishing diets over a 24-d period with four steps (6 d each) starting at experiment initiation. During each step, 10% corn replaced 5% corn silage and 5% wheat straw, while inclusion of supplement and Sweet Bran (0%, 20%, or 40%) remained constant (Table 1). The final treatment diets contained 15% corn silage and 5% supplement with Sweet Bran replacing corn in the diet (Table 2). SFC was processed to a flake density of 0.34 kg/L (26.5 lb/bu) at a commercial feedlot (Raikes Feedyard, Ashland, NE) and delivered to the research feedlot on a weekly basis. HMC was processed through a 122 cm roller mill (48-inch; Renn Mill Center Inc., Alberta, Canada), and stored in a covered concrete bunker for approximately 250 d before feeding. The moisture content of the HMC during the feeding period was 29.9%. All supplements were formulated to include 33 mg/kg DM of monensin (Rumensin; Elanco Animal Health; Greenfield, IN) and to provide 90 mg/steer DM of tylosin (Tylan; Elanco Animal Health). Ractopamine hydrochloride (Optaflexx; Elanco Animal Health) was fed the last 28 (medium and heavy blocks) or 42 (light block) d on feed to target 300  mg/steer daily. The ability for international trade agencies to detect very low levels of the compound has been used to deny trade shipments, therefore we use a 48-h voluntary withdrawal as recommended by the manufacturer. All treatments were treated the same, and there is no evidence any withdrawal of ractopamine impacts performance or carcass traits (Bryant et al., 2019). Concentration of crude protein (CP) in all diets was lower than expected. The corn fed in the study contained a lower concentration of CP than the previous year's samples used for diet formulation. Metabolizable protein (MP) and rumen degradable protein (RDP) balances were calculated using the beef cattle nutrient requirement model with inputs of average body weight and treatment DMI and ADG. The following adjustments were made from the National Academies of Science, Engineering, and Medicine (NASEM, 2016) model: (1) 8% microbial efficiency, (2) 40%, 55%, 40%, 75%, and 76% RDP for DRC, HMC, SFC, Sweet Bran, and corn silage, and (3) 90% rumen undegradable protein digestibility (RUPd) digestibility for corn ingredients, 80% RUPd for Sweet Bran, and 35% RUPd for corn silage.Net energy for maintenance and net energy for gain for all treatments were calculated using a generalized quadratic solution based on intake and performance by cattle, as suggested by Galyean (2017) using NRC equations (NRC, 1996). These equations calculate net energy for maintenance and net energy for gain using initial shrunk BW (pen average), final shrunk BW (pen average), shrunk BW at target endpoint (heaviest pen within a block), DMI (pen average), and ADG (pen average).

**Table 1.** Dietary composition of adaptation diets (DM)

	Diets^b^				
	Step 1	Step 2	Step 3	Step 4	Finisher
*0% Sweet Bran*					
Corn^a^	40	50	60	70	80
Sweet Bran	0	0	0	0	0
Corn silage	35	30	25	20	15
Wheat straw	20	15	10	5	0
Supplement	5	5	5	5	5
*20% Sweet Bran*					
Corn^a^	20	30	40	50	60
Sweet Bran	20	20	20	20	20
Corn silage	35	30	25	20	15
Wheat straw	20	15	10	5	0
Supplement	5	5	5	5	5
*40% Sweet Bran*					
Corn^a^	0	10	20	30	40
Sweet Bran	40	40	40	40	40
Corn silage	35	30	25	20	15
Wheat straw	20	15	10	5	0
Supplement	5	5	5	5	5

^a^Corn is either 100% steam-flaked corn (SFC) or 2/3 high-moisture corn (HMC) and 1/3 dry-rolled corn (DRC).

^b^Each step was fed for 6 d.

**Table 2.** Dietary treatment composition and chemical analysis (%, DM basis) for finishing steers fed steam-flaked corn (SFC) or high-moisture and dry-rolled corn (HMC:DRC) with 0%, 20%, or 40% Sweet Bran

	Treatment^a^					
	SFC			HMC:DRC		
Ingredient	0	20	40	0	20	40
Steam-flaked corn	80	60	40	—	—	—
High-moisture corn	—	—	—	53.33	40	26.67
Dry-rolled corn	—	—	—	26.67	20	13.33
Sweet Bran	0	20	40	0	20	40
Corn silage	15	15	15	15	15	15
Supplement^b^						
Fine ground corn	1.32	2.39	2.96	1.32	2.39	2.96
Limestone	1.66	1.59	1.52	1.66	1.59	1.52
Tallow	0.125	0.125	0.125	0.125	0.125	0.125
Urea	1.5	0.5	0	1.5	0.5	0
Salt	0.3	0.3	0.3	0.3	0.3	0.3
Vitamin A-D-E premix^c^	0.015	0.015	0.015	0.015	0.015	0.015
Trace mineral premix^d^	0.05	0.05	0.05	0.05	0.05	0.05
Rumensin 90 premix	0.017	0.017	0.017	0.017	0.017	0.017
Tylan 40 premix	0.009	0.009	0.009	0.009	0.009	0.009
*Chemical composition* ^e^						
Organic matter, %	96.2	94.9	93.7	95.6	94.4	93.3
Neutral detergent fiber, %	11.9	17.2	22.5	13.5	18.4	23.4
Acid detergent fiber, %	5.76	7.21	8.65	6.05	7.43	8.80
Crude protein, %	12.2	12.5	14.6	12.6	13.0	14.8
Starch, %	66.2	53.6	40.6	61.0	49.7	38.0
Calcium, %	0.80	0.80	0.79	0.77	0.78	0.78
Phosphorus, %	0.19	0.40	0.54	0.30	0.44	0.58
RDP, %^f^	7.80	7.92	9.41	8.73	8.60	9.87
NEm, Mcal/kg^g^	2.17	2.16	2.13	2.04	2.06	2.07
NEg, Mcal/kg^g^	1.50	1.49	1.46	1.39	1.41	1.41
MP balance^h^, g/d	74	74	103	161	156	155
RDP balance^h^, g/d	39	85	288	42	74	279

^a^Treatments included SFC 0: steam-flaked corn with 0% Sweet Bran, SFC 20: steam-flaked corn with 20% Sweet Bran, SFC 40: steam-flaked corn with 40% Sweet Bran, HMC:DRC 0: high-moisture corn/dry-rolled corn with 0% Sweet Bran, HMC:DRC 20: high-moisture corn/dry-rolled corn with 20% Sweet Bran, and HMC:DRC 40: high-moisture corn/dry-rolled corn with 40% Sweet Bran.

^b^Supplement fed at 5% of dietary DM for all treatments.

^c^Premix contained 30,000 IU vitamin A, 6,000 IU vitamin D, and 7.5 IU vitamin per gram.

^d^Premix contained 6.0% Zn, 5.0% Fe, 4.0% Mn, 2.0% Cu, 0.29% Mg, 0.2% I, and 0.05% Co.

^e^Analyzed composition from a commercial laboratory (Ward Laboratories, Kearney, NE).

^f^The following RDP values were assumed: 40%, 55%, 40%, 75%, and 76% RDP for DRC, HMC, SFC, Sweet Bran, and corn silage.

^g^Values for treatment diets were calculated from NASEM (2016) tabular NE values.

^h^Values based on the Beef Cattle Nutrient Requirement Model (BCNRM) using average body weight and treatment dry matter intake and ADG.

Feed bunks were assessed at approximately 0600 h and managed for trace (≤0.2 kg/steer) amounts of feed remaining in the bunk each morning at the time of feeding to achieve ad libitum feed intake. Cattle were fed once daily between 0700 and 1000 hours with a truck-mounted mixer and delivery unit (Roto-Mix; Dodge City, KS). Each treatment diet was fed in the same order each day, however the pen order within each treatment was rotated daily. Over the feeding period, the standard deviation in feeding time for an individual pen was 44 min. When needed, feed refusals were removed from the feed bunks, weighed, subsampled, and dried in a forced-air oven at 60 °C (model LBB2-21-1; Despatch Industries; Minneapolis, MN) for 48 h to determine DM (AOAC, 1999, method 943.01) and calculate refusal DM weight. Ingredient samples were sampled weekly for DM analysis and as-is ingredient inclusions were adjusted weekly. At the end of the trial, weekly ingredient samples were composited by month and sent to a commercial laboratory (Ward Laboratories, Kearney, NE) to be analyzed for DM (Gales, 1990), total starch (AOAC INTERNATIONAL, 2000), CP (LECO Co.), neutral and acid detergent fiber (ADF and NDF; Mertens, 1992; ANKOM Technology, 1998), and minerals (Campbell and Plank, 1991; Kovar, 2003).

The medium and heavy blocks were shipped on November 3, 2020 (154 d on feed). The light block was shipped 2 wk later to achieve similar fat thickness on November 17, 2020 (168 d on feed). On the day of shipping, 50% of the previous day’s DM was offered. Steers were shipped in the evening and harvested the following morning at a commercial abattoir (Greater Omaha; Omaha, NE). On the day of harvest, kill order using panel tag IDs, and hot carcass weight (HCW) were recorded, and carcass-adjusted final BW was calculated using a common 63% dressing percentage. Carcass-adjusted final BW was used to determine ADG and feed efficiency (gain:feed). On the day of harvest, liver abscess scores were recorded immediately after evisceration. The following liver scoring system was used: 0 for no abscesses, A- for one or two small abscesses, A for two to four small active abscesses, and A + for 1 or more active abscesses (Brink, 1990). Liver abscesses were then combined to determine the total proportion of liver abscesses per treatment. Following a 48 h-chill, USDA marbling score, 12th rib fatness thickness, and *longissimus* muscle (LM) area were recorded from an in-plant camera system. Yield grade was calculated using the USDA YG equation:

$\begin{array}{llll} & YG=2.5+(6.25\times 12th\;rib\;fat,cm)\; & \;+\;(0.2\times 2.5\left[2.5\;assumed\;average\;steer\;KPH,\%\right])\; & \;+\;(0.0017\times HCW,kg)-(2.06\times LM\;area,cm^{2}); \end{array}$ USDA, 1997).

### Statistical Analysis

Data were analyzed using the MIXED procedure of SAS (SAS Inst., Inc., Cary, NC) as a generalized block design with pen as the experimental unit and block as a fixed effect. Data were tested for a linear and quadratic interaction between treatment factors using covariate regression (Littell et al., 2002). If an interaction was observed, then simple effects (linear or quadratic) of Sweet Bran inclusion were evaluated within each corn processing method. If no interaction was observed, then main effects of corn processing method and Sweet Bran inclusion were evaluated. Liver abscesses were analyzed using the GLIMMIX procedure of SAS as a binomial distribution evaluating the presence or absence of liver abscesses. Arithmetic means are presented due to unbalanced replications across blocks for initial BW. Probabilities less than or equal to alpha (*P *≤ 0.05) were considered statistically significant, with tendencies acknowledged at *P* > 0.05 and *P *≤ 0.10.

## Results and Discussion

### Interaction of Corn Processing Method and Sweet Bran Inclusion

**Figure 1.**
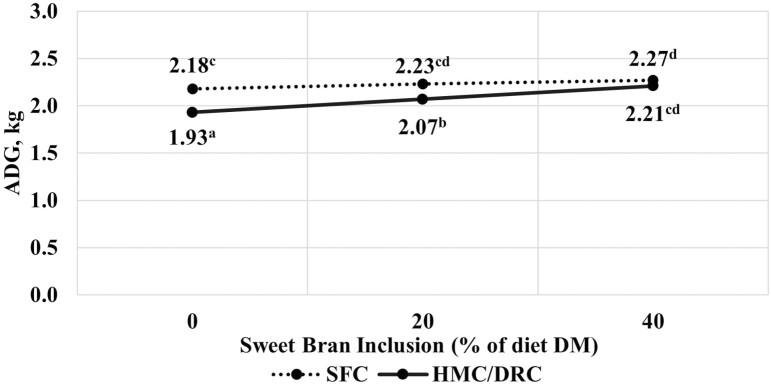
Effect of corn processing method and Sweet Bran inclusion on ADG. Corn processing methods include steam flaking (SFC) or a blend of 2/3 high-moisture corn and 1/3 dry-rolled corn (HMC:DRC). The linear interaction of corn processing method and Sweet Bran was significant (*P* < 0.01; SEM = 0.03).

There were no quadratic interactions between corn processing method and Sweet Bran inclusion or quadratic main effects of Sweet Bran inclusion (*P* > 0.22). A corn processing × Sweet Bran interaction was observed for ADG (*P* < 0.01; Table 3; Figure 1). In both SFC and HMC: DRC-based diets, ADG increased linearly with increasing concentration of Sweet Bran (*P* < 0.05). However, the interaction occurred (*P* < 0.01) because ADG increased at a greater rate for HMC: DRC-based diets (1.93 to 2.21 kg/d for 0% to 40% Sweet Bran, respectively) compared to SFC-based diets (2.18 to 2.27 kg/d for 0% to 40% Sweet Bran, respectively). Accordingly, while the ADG of steers fed SFC was 13% greater than steers fed HMC: DRC without Sweet Bran (*P *< 0.01), there was no difference in ADG due to corn processing method at 40% Sweet Bran (*P* = 0.30). In two separate trials, Scott et al. (2003) compared diets containing DRC or SFC with 0% or 32% Sweet Bran and observed a similar response for ADG to the current study. The ADG of steers fed SFC was on average 3.8% greater than steers fed DRC without Sweet Bran, but no difference in ADG was observed at 32% Sweet Bran.

**Table 3.** Simple Effects of carcass-adjusted performance of cattle fed steam-flaked corn (SFC) or a combination of high-moisture and dry-rolled corn (HMC:DRC) with 0%, 20%, or 40% Sweet Bran (SB)^1^

Treatment^2^							*P*-value^3^			
	SFC			HMC:DRC				Corn	SB	Corn × SB linear
	0	20	40	0	20	40	SEM			
*Performance*										
Initial BW, kg	362	362	362	362	362	362	5.3	0.81	0.34	0.77
Final BW^4^, kg	709^c^	716^c^	723^c^	668^a^	690^b^	712^c^	4.9	<0.01	<0.01	<0.01
DMI, kg/d	12.1	12.5	12.8	12.0	12.5	13.0	0.14	0.89	<0.01	0.14
ADG, kg	2.18^c^	2.23^cd^	2.27^d^	1.93^a^	2.07^b^	2.21^cd^	0.030	<0.01	<0.01	<0.01
Gain:Feed	0.181^c^	0.179^c^	0.177^c^	0.161^a^	0.166^ab^	0.170^b^	0.0020	<0.01	0.24	<0.01
NEm, Mcal/kg^5^	2.02	1.98	1.98	1.85	1.88	1.90	0.018	<0.01	0.63	0.02
NEg, Mcal/kg^5^	1.36	1.33	1.32	1.21	1.23	1.26	0.016	<0.01	0.63	0.01
*Carcass characteristics*										
HCW, kg	446^c^	450^c^	455^c^	421^a^	435^b^	449^c^	3.1	<0.01	<0.01	<0.01
LM area, cm^2^	96.8	97.4	98.1	93.5	94.8	96.1	1.03	<0.01	0.07	0.60
12th rib fat, cm	1.55	1.60	1.68	1.47	1.55	1.60	0.05	0.10	0.02	0.92
Marbling^6^	512	520	528	486	488	490	11.3	<0.01	0.42	0.60
Calculated YG^7^	3.45	3.51	3.57	3.31	3.43	3.54	0.08	0.27	0.05	0.57
Liver abscesses, %	41.6	29.1	43.6	58.8	55.0	48.8	5.6	<0.02	0.33	0.17

^1^Arthmetic means are reported.

^2^Treatments included SFC 0: steam-flaked corn with 0% Sweet Bran, SFC 20: steam-flaked corn with 20% Sweet Bran, SFC 40: steam-flaked corn with 40% Sweet Bran, HMC:DRC 0: high-moisture corn/dry-rolled corn with 0% Sweet Bran, HMC:DRC 20: high-moisture corn/dry-rolled corn with 20% Sweet Bran, and HMC:DRC 40: high-moisture corn/dry-rolled corn with 40% Sweet Bran.

^3^Corn = *P*-value for the main effect of corn processing method, SB = *P*-value for the main effect of Sweet Bran inclusion, Corn × SB Linear = *P*-value for the linear interaction between corn processing method and Sweet Bran inclusion.

^4^Calculated on a carcass-adjusted basis using a common dressing percentage (63%).

^5^Calculated using a generalized quadratic solution based on intake and performance by cattle, suggested by Galyean.

^6^Marbling score 300 = slight, 400 = small, 500 = modest, etc.

^7^ $\begin{array}{ll} & YG=2.5+(6.25\times 12th\;rib\;fat,cm)+(0.02\times 2.5\;\left[2.5\;assumed\;average\;steer\;KPH,\%\right]) \end{array}$ $\begin{array}{l} +\;(0.0017\times HCW,kg)-(2.06\times LM\;area,cm^{2}) \end{array}$

^abcd^Means in a row with different superscripts are different (*P* ≤ 0.05).

**Figure 2.**
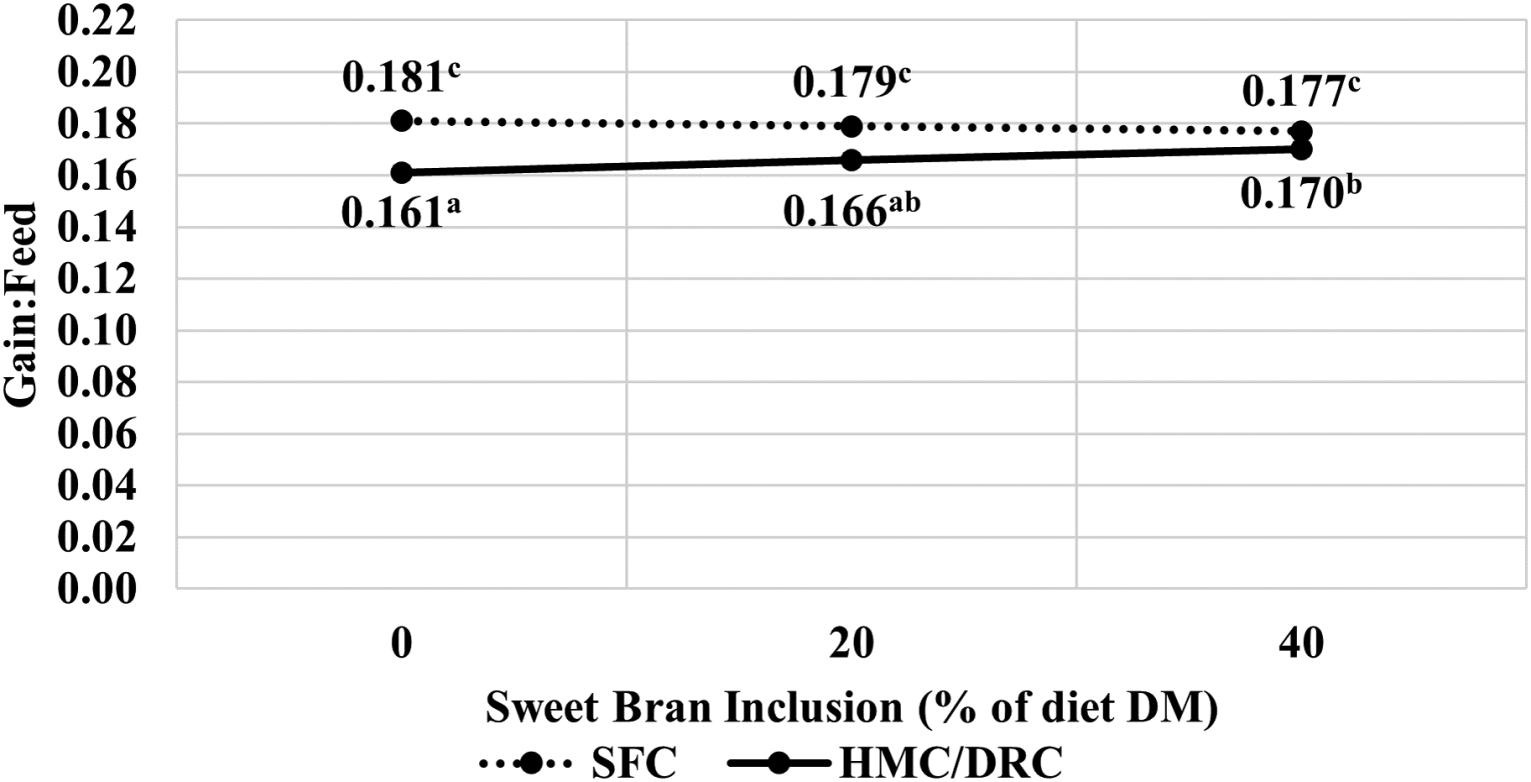
Effect of corn processing method and Sweet Bran inclusion on gain to feed ratio. Corn processing methods include steam flaking (SFC) or a blend of 2/3 high-moisture corn and 1/3 dry-rolled corn (HMC:DRC). The linear interaction of corn processing method and Sweet Bran was significant (*P* < 0.01; SEM = 0.002).

A corn processing × Sweet Bran interaction was also observed for feed efficiency (G:F; *P* < 0.01; Figure 2). The feed efficiency of steers fed SFC did not change with increasing Sweet Bran concentrations (*P* = 0.19); however, G: F improved linearly for steers fed HMC: DRC with increasing Sweet Bran concentrations (*P* < 0.01). There was a 12.4% improvement in G:F when feeding SFC compared to HMC: DRC, which is consistent with previous research that have shown improvements of greater than 9.4% for G:F (Zinn et al., 1998; Brown et al., 2000; Corrigan et al., 2009). While the G:F for SFC remained greater than HMC:DRC when 40% Sweet Bran was fed (*P = *0.04), the improvement of SFC over HMC:DRC in G:F was only 5.3% when 40% Sweet Bran was fed compared to 12.4% when no Sweet Bran was fed.

The interaction of corn processing and Sweet Bran inclusion may be further explained by evaluating the response to both DMI and ADG. In the current study, DMI increased linearly as Sweet Bran increased in the diet from 0% to 40% regardless of corn processing method, and as a result, ADG also improved linearly in SFC and HMC:DRC-based diets. In SFC-based diets, the magnitude of improvement for DMI and ADG were similar, resulting in no change in G:F in these diets. This is consistent with the observations of Scott et al. (2003) who also observed an increase in DMI and ADG with no change in G:F in two separate trials feeding SFC with (22 or 32%) or without Sweet Bran. Macken et al. (2004) also observed a tendency for an increase in DMI in diets containing 0%, 10%, 20%, 25%, 30%, and 35% Sweet Bran similar to both the current study and Scott et al. (2003). However, there were no improvements in ADG or G:F in the study by Macken et al. (2004). In contrast, Block et al. (2005) reported a quadratic response for DMI, ADG, and G:F in SFC diets containing 0%, 20%, 30%, and 40% Sweet Bran with ADG and G:F optimizing in the range of 20% to 30% inclusion. The quadratic response in DMI as Sweet Bran increased from 0% to 40% was driving the response in cattle performance observed by Block et al. (2005) and is inconsistent with previous research, which observed a linear increase in DMI. Domby et al. (2014) also observed a quadratic response for G:F in diets containing 0%, 15%, and 30% Sweet Bran in SFC-based diets, suggesting feed efficiency is optimized between 15% and 20%, similar to Block et al. (2005). However, no change in DMI was observed, which contradicts the current study and previous studies (Scott et al., 2003; Macken et al., 2004). Numerically, Domby et al. (2014) observed a quadratic response for ADG with increasing Sweet Bran concentration, but the lack of statistical significance agrees with no improvement in ADG, similar to Macken et al. (2004). In HMC and DRC-based finishing diets, Bremer et al. (2008) summarized six studies feeding 0% to 40% Sweet Bran and reported a linear improvement in ADG and G:F, which agrees with the linear improvement observed in the current study.

It should be acknowledged that the improvement in performance with increasing Sweet Bran concentration in the current study may be due to the increased CP concentration in the diet. The SFC and HMC:DRC-based diets were formulated to contain 12.8% CP, respectively. Subsequent analysis of the diet ingredients showed the CP content of the SFC and HMC:DRC-based diets without Sweet Bran were 12.2% and 12.6%, respectively (Table 2). This CP concentration is below the industry standards described by Samuelson et al. (2016) and was the result of lower-than-expected CP concentrations in the corn fed in the study. However, if the improvement in performance were due to a protein response, it would be expected that steers fed SFC would be more deficient than steers fed HMC:DRC. Yet the G:F of steers SFC-based diets did not increase as the concentration of Sweet Bran increased, as would be expected. Additionally, a linear improvement in G:F for HMC:DRC-based diets was observed with increasing concentrations of Sweet Bran. If the improvement in performance were due to a protein response, the addition of Sweet Bran would be expected to result in a quadratic change in G:F, with maximum efficiency observed where the protein requirement was met. Based on this information, we conclude the performance responses in the current study are not a CP response. Additionally, beef cattle nutrient requirement model with the adjustments described above, all 6 treatment diets, even with the lesser-than-expected CP concentration in corn, suggest excess MP while RDP is sufficient. MP in excess can be recycled to meet RDP requirements. We acknowledge that based on the % RDP of the diet (Table 3), according to Cooper et al. (2002), steers would be deficient in RDP. Cooper et al. (2002) recommend 6.3%, 10%, and 8.5% RDP for DRC, HMC, and SFC, respectively.

**Figure 3.**
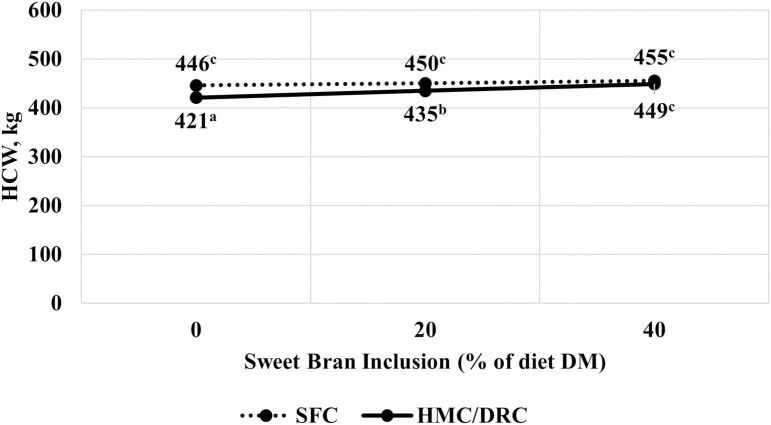
Effect of corn processing method and Sweet Bran inclusion on hot carcass weight (HCW). Corn processing methods include steam flaking (SFC) or a blend of 2/3 high-moisture corn and 1/3 dry-rolled corn (HMC:DRC). The linear interaction of corn processing method and Sweet Bran was significant (*P* < 0.01; SEM = 3.1).

**Figure 4.**
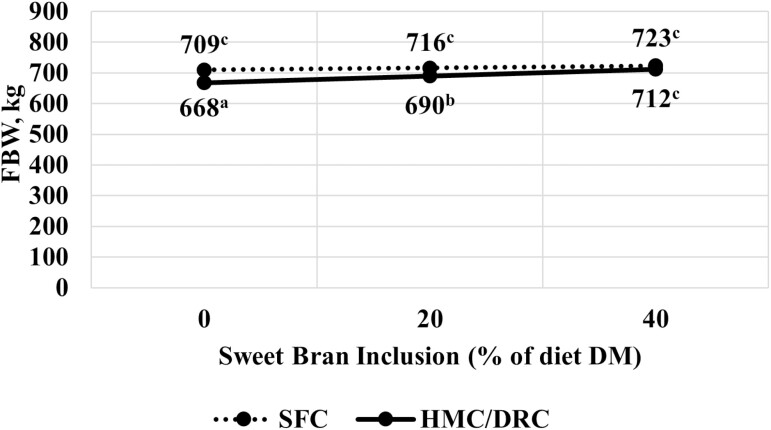
Effect of corn processing method and Sweet Bran inclusion on carcass-adjusted final body weight (FBW). Corn processing methods include steam flaking (SFC) or a blend of 2/3 high-moisture corn and 1/3 dry-rolled corn (HMC:DRC). The linear interaction of corn processing method and Sweet Bran was significant (*P* < 0.01; SEM = 4.9).

There was a corn processing × Sweet Bran interaction for HCW and carcass-adjusted final BW (*P* < 0.01; Figures 3 and 4). Steers fed SFC had greater HCW and carcass-adjusted final BW than cattle fed HMC: DRC (*P* < 0.01). As Sweet Bran concentration increased, HCW and carcass-adjusted final BW tended (*P* = 0.06) to increase for cattle-fed SFC and significantly increased (*P* < 0.01) for cattle-fed HMC: DRC. As a result, HCW and carcass-adjusted final BW were not different between the two corn processing methods at 40% Sweet Bran (*P* = 0.28).

No interactions were observed for DMI, LM area, rib fat depth, marbling, calculated yield grade, or liver abscesses (*P* ≥ 0.14), so main effects of corn processing method or Sweet Bran inclusion will be discussed.

### Main Effects of Corn Processing on Performance and Carcass Characteristics

Steers fed SFC had a larger LM area than steers fed HMC: DRC (*P* < 0.01; Table 3). Steers fed SFC tended to have greater rib fat depth (*P* = 0.10) than steers fed HMC: DRC. Accordingly, steers fed SFC had a greater degree of marbling compared to steers fed HMC:DRC (*P* < 0.01). Impacts on carcass traits likely reflect changes in ADG as cattle-fed SFC generally gained faster. Steers fed HMC: DRC had a greater prevalence of liver abscesses compared to steers fed SFC (*P* < 0.02). It is unclear why liver abscess rates were abnormally high as all steers were fed tylosin in this study.

### Main Effects of Sweet Bran Inclusion on Performance and Carcass Characteristics

DMI increased as Sweet Bran increased in the diet regardless of corn processing method (*P* < 0.01). The linear effect of increasing DMI as inclusion of Sweet Bran increased is consistent with previous research in SFC (Scott et al., 2003 and; Macken et al., 2004) and HMC and DRC-based finishing diets (Bremer et al., 2008). A linear increase in 12th rib fat was observed with fat increasing as Sweet Bran increased in the diet (*P* = 0.02), which led to a linear increase in calculated yield grade (*P* = 0.05). Feeding diets with Sweet Bran increased ADG resulting in a more rapid deposition of rib fat (Bremer et al., 2008). Similarly, Scott et al. (2003) reported greater yield grade and 12th rib fat in cattle fed 22% Sweet Bran and Buckner et al. (2007) reported greater yield grade when cattle were fed 30% Sweet Bran. As Sweet Bran increased in the diet, LM area also tended to increase linearly (*P* = 0.07). In contrast to the current study, differences in LM area have not typically been observed with varying concentrations of Sweet Bran (Macken et al., 2004; Block et al., 2005).

### Implications

These data suggest there is flexibility in feeding Sweet Bran in SFC-based finishing diets. While gain is maximized at 40% Sweet Bran, feed efficiency is similar across Sweet Bran inclusions. In HMC:DRC-based finishing diets, gain, and feed efficiency were both maximized at 40% Sweet Bran. Feeding Sweet Bran in HMC:DRC-based finishing diets make these diets more competitive with SFC-based finishing diets thereby allowing producers without steam-flaking capabilities the ability to achieve similar gains and more similar feed efficiency to SFC-based diets.
